# Determination of cyanide in urine and saliva samples by ion chromatography with pulsed amperometric detection

**DOI:** 10.1007/s00706-017-1977-x

**Published:** 2017-06-23

**Authors:** Ewa Jaszczak, Sylwia Narkowicz, Jacek Namieśnik, Żaneta Polkowska

**Affiliations:** Department of Analytical Chemistry, Chemical Faculty, Gdansk University of Technology, Narutowicza Str. 11/12, 80-233 Gdansk, Poland

**Keywords:** Cyanide, IC-PAD, Tobacco smoke, Urine, Saliva

## Abstract

**Abstract:**

Commonly known as a highly toxic chemical, cyanide is also an essential reagent for many industrial processes. It naturally occurs in plant seeds as cyanogenic glycosides. Another relatively common mode of cyanide exposure is inhalation of environmental tobacco smoke. This study concerns importance to determine cyanide ion in human biological samples. Urine and saliva samples were collected healthy volunteers exposed to tobacco smoke (active smokers) and environmental tobacco smoke (passive smokers). Chromatographic separation was achieved with an anion-exchange column and separated ions were detected by a pulsed amperometric detector. The method produced linear response in a specific concentration range of cyanide ion. The limit of detection was estimated at 0.1  and 0.5 µg/dm^3^ for urine and saliva samples, respectively. Cyanide ion concentrations in samples ranged from not detected (below LOD) to 12.88 µg/dm^3^. The comparison of results of biological samples analyses shows an increasing trend in cyanide concentration that may suggest that environmental tobacco smoke might have an impact on human health.

**Graphical abstract:**

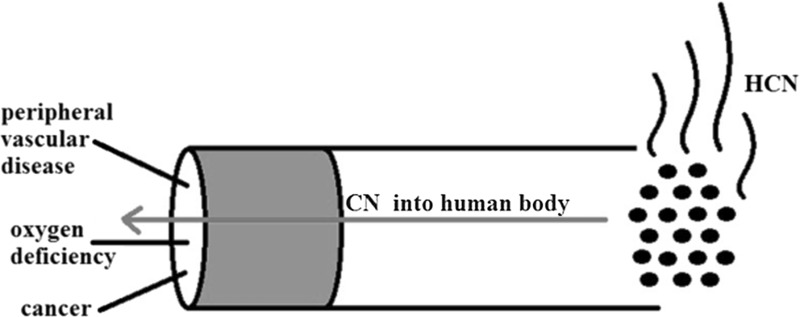

## Introduction

Cyanides are used in various areas of industrial activity, including mining and chemical industry. HCN is also formed directly in fossil fuel combustion from gasoline and diesel vehicles [1, 2]. Natural sources of cyanide are cyanogenic glycosides found in the seeds of plants such as apricots, peaches and plums [3–5]. Furthermore, cyanides may also be present in drinking water, soil, and air in previously mentioned industrial activities. The presence of cyanide ions in the air is the result of the issuance of car exhaust and fires [6]. Human exposure to cyanide is also associated with tobacco smoking. Vulnerable people include both active and passive smokers. They are in contact with tobacco smoke constituents present in air (indoor air, atmospheric air). This mixture is known as environmental tobacco smoke (ETS) [7].

**Fig. 1:**
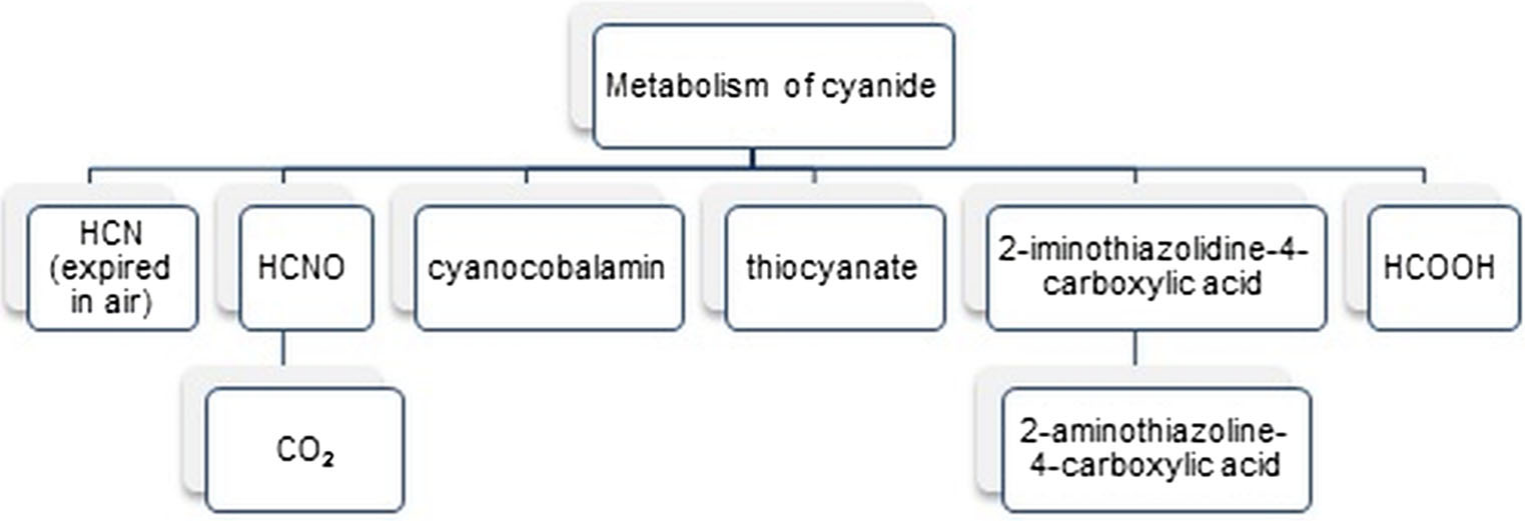
Basic processes involved in the metabolism of cyanide

The toxic effect of cyanide is based on a combination of trivalent cation iron cytochrome a3, an integral component of the enzyme cytochrome oxidase located in the mitochondria of liver. The combination of cyanide ion with this enzyme results in inhibition of cell respiration and severity of anaerobic glycolysis. The half-life of cyanide ions in the body is about 2 h and then they are metabolized to a less toxic form, excreted with body fluids (Fig. 1) [8].

Prolonged exposure to ETS components including cyanide leads to the weakening of the body, the occurrence of many diseases and even death. It is estimated that the lethal dose for humans is 1.5 mg/kg [9]. In view of the toxicity of cyanide, it is necessary to monitor the levels of cyanide content in both biological and environmental materials. Human biological materials are an excellent source of information on environmental pollution and its impact on human health and life [10–12]. The recent methods cover both established and emerging analytical disciplines such as spectrophotometry, capillary electrophoresis with optical absorbance detection, atomic absorption spectrometry, electrochemical methods (potentiometry/amperometry/ion chromatography-pulsed amperometry), and gas chromatography [13–15]. With the development of ion chromatography as a precise and simultaneous analytical technique, successful determination of cyanide ion in biological sample such as urine has been achieved.

The studies described in this article mainly focus on the impact of environmental tobacco smoke on cyanide concentration in human biological samples which in turn has an impact on human health. Moreover, cyanide ions may be biomarkers of exposure to tobacco smoke components; therefore, the proposed method can also be applied for medical use.

## Results and discussion

The procedure for determining cyanide ion in biological samples was validated to ensure the appropriate level of quality control and quality assurance of measurements. Calibration curve was generated in the range from 0.3 to 150 µg/dm^3^ NaCN by plotting the ratio of the peak areas against the concentrations of respective standards. The determined calibration curve was used to calculate the concentration of analytes in the urine samples. The limit of determination was obtained on the basis of the signal-to-noise ratio for blank samples from control group (very low analyte concentration). The precision of developed procedure was expressed as the coefficient of variation calculated for three replicates (Table 1).

**Table 1:** Technical specifications and metrological characteristics of IC-PAD

Sample	Measurement range/µg dm^−3^	LOD/µg dm^−3^	*R*	SD	CV/%	Recovery/%
Urine	1–100	0.1	0.922	0.003	1.63	80
Saliva	5–100	0.5	0.994	0.05	1.84	113

Urine samples were collected from the volunteers who were students from Chemical Faculty of Gdansk University of Technology. The study was approved by ethics Committee of the Medical University of Gdansk. Tests were performed in samples from volunteers who constituted three groups: smokers, passive smokers, and members of control group (Table 2). All samples were collected in the morning and all subjects were aged 21–30 years. They were not on special diet before the tests, but some of volunteers declared diet rich in dairy products, broccoli, and almonds. The reason for cyanide poisoning, as a consequence of food consumption, is cyanogenic glycoside in plants. The most common cyanogenic glycoside is amygdalin that can be found in seeds, pips, and kernel of fruit such as apples, peaches, almonds, cherries, plums, and apricots. A diet rich in these products may affect the concentration of the cyanide ions in samples of biological material. Taking hormone medications for some women did not affect the result of the analysis and only drugs for high blood pressure can release cyanide group [16]. Nobody declared exposure to fire smoke.

**Table 2:** Information about 104 volunteers’ samples

	Number of			BMI		Time of smoking	Average number of smoked cigarettes	Passive smoking at work	Passive smoking at home
	Subjects	Women	Men	Women	Men				
Urine									
Smokers	26	16	10	22.2	24.9	In the evening	6–10	Yes	Yes
Passive smokers	25	13	12	23.01	22.32	n/a	n/a	Yes	Yes
Control group	16	10	6	20.7	22.2	n/a	n/a	No	No
Saliva									
Smokers	11	8	3	22.7	24.2	In the evening	1–5	No	Yes
Passive smokers	16	11	5	22.4	23.1	n/a	n/a	No	Yes
Control group	10	7	3	21.8	23.4	n/a	n/a	No	No

**Fig. 2:**
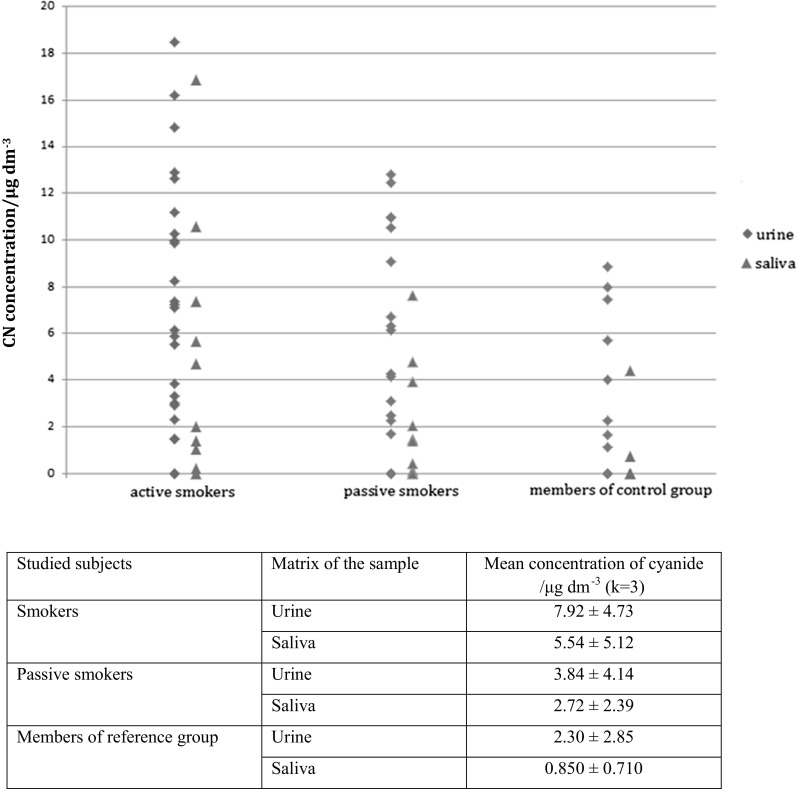
Cyanide concentrations for urine and saliva samples of smokers, passive smokers, and members of reference group

The concentrations of cyanide ion in urine samples are listed in Fig. 2. The concentration of cyanide is higher in urine samples than in saliva samples. The half-life of cyanide ions in the body is about 2 h, then they are excreted through body fluids. Samples of saliva were collected in the morning, just before lighting a cigarette by an active smoker. One person from the group of smokers declared occasional smoking e-cigarettes, but the analysis result was below LOD of the analytical procedure. Tobacco smoking is the leading cause of introducing cyanide ions into human body. Another daily source is cyanogenic glycosides in plants usually at low concentrations and it is a rare source of contamination. Although none of the donors was the victim of a fire, one of the non-smoking donors whom the result of the analysis confirmed the presence of cyanide in the urine and he admitted in a survey that the previous day he took part in a meeting with barbeque. The estimated lethal dose for an adult human is 1.5 mg CN^−^/kg of body weight. Symptoms of severe poisoning by inhalation are observed from 53 mg HCN/m^3^, while the lethal dose ingested with food is approximately 200–300 mg. Although the urine analysis results are less than a lethal dose, there might be potential risk for human health. Inhalation of hydrogen cyanide gas causes a distinct slowing of heart rate with further palpitations. The gastrointestinal effects resulting from cyanide exposure, like vomiting, are provoked by irritation of gastric mucosa, when the gas is being breathed in. Chronic exposure of cyanide to human has produced neurological effects such as hemianopia [9, 16].

## Conclusions

The applicability of ion chromatography with pulsed amperometric detection for the determination of cyanide in two matrices was evaluated in this study. Sample preparation included the stabilization of the sample pH and the use of Cartridge II H filter. The method was effective in reducing the sample background and removing interferences in urine and saliva samples. The highest concentrations of cyanide ion in the case of urine samples were detected in the group of actively smoking volunteers. Cyanide ions may be biomarkers of exposure to environmental tobacco smoke. This method offers high sensitivity and short analysis duration. The proposed method can be used for the routine analysis of cyanide in biological samples.

## Experimental

All reagents and chemicals (50% NaOH and NaCN) were purchased from Sigma-Aldrich. Deionized water was obtained from Millipore Gradient A10 (resistivity 18.2 Ω cm at 25 °C) water purification system (Millipore, Bedford, USA). The calibration solutions were prepared by dissolving stock solution with 100 mM NaOH. To obtain stock solution of 1000 mg/dm^3^ NaCN, 0.0377 g of this salt in powder was placed in polyethylene volumetric flask and filled up by 100 mM NaOH to a total weight of 20 g. To prepare working standards of cyanide from 1 mg intermediate standard, an appropriate amount of NaCN was diluted by 100 mM to total weight of 20 g.

### Sample preparation

Cyanide is reactive and unstable; therefore, urine samples should be stabilized as soon as is possible by adding 100 mM NaOH to make pH around 12. It is necessary to add sodium hydroxide to stabilize the forms of occurrence of cyanide. Urine sample was stored in refrigerator before analysis. The 5 cm^3^ of urine was used for analysis. To remove particles, sample was filtered through a Cartridge II H filter (Dionex, Sunnyvale, CA, USA). This filtrate was taken to be filtered through a 0.45-µm syringe filter. The resulting filtrate (1 cm^3^) was diluted (1:3) with deionized water and analyzed immediately. The analysis of each sample was repeated three times.

### Instrumentals

The parameters of the ion chromatograph (ICS 3000, Dionex, Sunnyvale, CA, USA) are collected in Table 3. The IC was performed with an anion guard column (Dionex IonPac AG15), an anion separator column (Thermo Scientific, Dionex IonPac AS15 analytical), and pulsed amperometric detection.

**Table 3:** Condition of IC-PAD analysis

Flow rate	1 cm^3^/min
Eluent	63 mM NaOH
Column temp.	30 °C
Tray temp.	10 °C
Inj. volume	10 mm^3^, full-loop injection
Electrodes	Reference: pH-Ag/AgCl electrode in AgCl mode
	Working: certified disposable Ag working electrode
Background	3–13 nC
Backpressure	≈110 psi
Noise	<7 psi
Run time	25 min
